# A Role for Exercise in Attenuating Unhealthy Food Consumption in Response to Stress

**DOI:** 10.3390/nu10020176

**Published:** 2018-02-06

**Authors:** Shina Leow, Ben Jackson, Jacqueline A. Alderson, Kym J. Guelfi, James A. Dimmock

**Affiliations:** 1 School of Human Sciences, The University of Western Australia, 35 Stirling Highway, Crawley, Perth, WA 6009, Australia; ben.jackson@uwa.edu.au (B.J.); jacqueline.alderson@uwa.edu.au (J.A.A.); kym.guelfi@uwa.edu.au (K.J.G.); james.dimmock@uwa.edu.au (J.A.D.); 2 Auckland University of Technology, Sports Performance Research Institute New Zealand (SPRINZ), Private Bag 92006, Auckland 1142, New Zealand

**Keywords:** psychological stress, exercise, physical activity, food choices, energy intake, appetite

## Abstract

It is well established that both acute and chronic stress can be detrimental to health and wellbeing by directly increasing the risk of several chronic diseases and related health problems. In addition, stress may contribute to ill-health indirectly via its downstream effects on individuals’ health-related behaviour, such as promoting the intake of unhealthy palatable foods high in fat and sugar content. This paper reviews (a) the research literature on stress-models; (b) recent research investigating stress-induced eating and (c) the potential physiological and psychological pathways contributing to stress-induced eating. Particular attention is given to (d) the role of physical exercise in attenuating acute stress, with exploration of potential mechanisms through which exercise may reduce unhealthy food and drink consumption subsequent to stressor exposure. Finally, exercise motivation is discussed as an important psychological influence over the capacity for physical exercise to attenuate unhealthy food and drink consumption after exposure to stressors. This paper aims to provide a better understanding of how physical exercise might alleviate stress-induced unhealthy food choices.

## 1. Introduction

Stress is associated with a multitude of harmful psychological and physiological effects, including worry, anxiety, increased blood pressure and inflammatory processes linked to atherosclerosis [1,2,3]. As well as influencing health outcomes directly, stress may also contribute to ill-health indirectly by influencing the consumption of unhealthy foods and drinks. Indeed, recent evidence indicates that, in some circumstances, people may respond to acute stress by increasing the consumption of unhealthy, energy-dense snack foods that are high in sugar and saturated and trans fats (i.e., “stress-induced eating”) (e.g., [4,5,6]). Regular consumption of these foods is associated with numerous health consequences including overweight/obesity, hypertension and hyperlipidemia—all well-established risk factors for cardiovascular disease [7,8,9]. In light of the significant impact of stress on health, it is not surprising that an abundance of research has been undertaken examining the efficacy of stress reduction techniques. Often, these techniques have focused on reducing the magnitude of immediate stress responses, rather than reducing negative downstream behavioural outcomes of stress. However, a holistic appreciation of the benefits of stress reduction techniques requires examination, not only of their attenuation of immediate psychological and physiological responses to stress but also their effects on health behaviour following stressor exposure.

It is well established that physical exercise is an often-used stress management technique that plays an important role in supporting a number of health and wellbeing outcomes [10,11,12]. However, despite there being some evidence that exercise undertaken prior to exposure to an acute stressful episode can down-regulate an individual’s acute stress response [13,14,15,16], the role of exercise in attenuating post-stressor eating behaviour remains unclear. Moreover, the extent to which exercise motivation—which is tied to individuals’ affective responses to exercise—moderates the impact of exercise on stress reactivity and post-stressor food choices, has not been explored. In this paper, we argue that exercise undertaken prior to an acute stressor is likely to reduce not only the magnitude of the immediate stress response but also subsequent intake of unhealthy foods and that these effects are most pronounced for those who possess high quality (i.e., autonomous) exercise motivation. In order to make this argument, we present (a) an overview of various stress models; (b) a review of recent research pertaining to stress-induced eating; (c) a summary of potential physiological and psychological pathways that contribute to stress-induced eating and (d) a discussion of how physical exercise may help to alleviate unhealthy eating following exposure to stressors.

## 2. Understanding “Stress”

Hans Selye, often referred to as the “father of stress”, defined stress as a nonspecific response of the body to any demand made upon it [17]. The stimulus that causes a stress response is termed a “stressor” [18] and these stressors may be classified as external or internal in nature. External stressors include stimuli such as major life changes (e.g., marriage, new home, death of a loved one), unpredictable events (e.g., car breakdown, unplanned pregnancy), or social demands (e.g., meeting new people). Internal stressors, on the other hand, refer to stimuli such as personal fears (e.g., public speaking, fear of flying), uncertainty (e.g., awaiting results of a medical test), or unrealistic perfectionistic expectations [19]. Acute stress is experienced when demands and pressures of the recent past and/or anticipated demands and pressures of the near future are present (i.e., recent and/or anticipated stressors), whereas chronic stress is associated with extended periods of unrelenting demands and pressures (i.e., long-term exposure to stressors) [19]. Individuals may respond to stressors differently depending on the type, nature and subjective interpretation of the stimuli and as a result, various stress models have been developed with the goal of better understanding the nature of the stress response. Two of the most influential stress models are the General Adaptation Syndrome (GAS; [17]) model and the Transactional Model [20] and in the material that follows, we provide a brief overview of these frameworks.

In the GAS model, Selye [17] proposed that different types of stressors result in a similar physiological response. The GAS theory consists of three phases, the alarm stage, resistance stage and exhaustion stage. In the alarm stage, the body responds with a burst of energy to deal with the presence of a stressor (acute in nature), also known as the “fight or flight” response [21]. In addition to an increase in heart rate, breathing rate and blood glucose levels, the adrenal glands release cortisol and adrenaline (catecholamine). After this initial response to the stressor, the resistance stage involves the body remaining on guard and the adrenal cortex continuing to release glucocorticoids (cortisol) to help the body react to the stressor until the stress is resolved, or the body can no longer resist and reserves are depleted. Lastly, the exhaustion stage occurs when the stressor continues without resolution for a sufficient time period or at a sufficient intensity, such that energy reserves are depleted. Some suggest that high levels and/or prolonged stress during this stage may leave the body vulnerable to physical and mental illness or disease [22]. Overall, the GAS model presents a biological explanation of how the body responds and adapts to the presence of stressors.

Other researchers have focused more closely on the psychological aspects or nature of stress. Lazarus and Folkman [20] claim stress does not directly result from stressors; but rather, emerges because of an individual’s inability to satisfy the demands that accompany a stressor. Within their Transactional Model, these scholars emphasize that “stress” occurs as a result of an imbalance between what a situation demands and the resources an individual possesses in relation to those demands. The Transactional Model consists of a two-stage cognitive appraisal process (i.e., the primary appraisal and the secondary appraisal). In the stage of the primary appraisal, the individual identifies how personally significant and threatening the stressor is and determines whether the stressor is a “threat”, “challenge”, or “harm-loss”. More specifically, individuals appraise the stressor as a “threat” when the anticipation of harm is imminent, a “challenge” when a person anticipates opportunity gains, or a “harm-loss” when psychological damage has already been experienced. Also, specific types of emotional reactions are proposed to be associated with each different primary appraisal classification [23]. For instance, an appraisal of a stressor as a threat might stimulate the individual to feel worried, fearful, or anxious; a challenge appraisal might evoke confidence, eagerness and hopefulness; and, a harm-loss appraisal might elicit anger, disgust, disappointment, or sadness. In the secondary appraisal, which operates independently from but may happen contemporaneously with, the primary appraisal, individuals assess their coping resources and their ability to meet the demands of the stressor. As evidence of the inter-relationships between primary and secondary appraisals, it is recognized that in some situations, the secondary appraisal may actually become the cause of a primary appraisal. For example, a secondary appraisal of inadequate coping resources might allow an individual to appraise (or possibly, re-appraise) a stressor as more of a threat than a challenge.

Of note, most often in the literature, the term “stress” is not used to describe specific components of the transaction between the person and the environment [24] but rather to represent the overall process incorporating stressors, stress demands, appraisals and stress responses (physiological and psychological). Hence, in this review, unless referring to a specific component of the stress model, we use the term “stress” to represent the overall process.

## 3. The Problem—Distress

Exposure to stressors and acute stress, is inevitable and it is common to experience stress as we strive to best use our resources to meet the requirements of our ever-demanding environment [25]. However, as discussed previously, exposure to stressors need not always be considered a “bad” thing. “Eustress”, for example, is a term introduced by Selye to represent “positive stress” and was later defined by Lazarus [26] as a positive stress response when a stressor is perceived as a challenge (i.e., primary appraisal of the Transactional Model). Indeed, in very early work, the concept of eustress was outlined within the Yerkes-Dodson curve [27]. The Yerkes-Dodson curve indicates that as stress levels increase, individuals’ performance in response to stress may also increase, to the point that an optimal level of stress is reached. Up to the point of optimal stress, individuals are proposed to adopt challenge appraisals and perceive that they have the resources to cope with the stressors. There is empirical support for this notion where some level of stress can drive perseverance in situations such as meeting work deadlines or striving to perform at one’s best in sport [28,29]. Similarly, recent research has demonstrated that when stressors are appraised in a positive (compared to a more distressing) light, individual performance may improve in social evaluative settings and public speaking tasks [30,31].

There are instances, however, in which exposure to stressors stimulates a negative stress response. In these situations, “distress” reflects a negative stress response, where the stressor is appraised as a threat or a harm-loss. According to the Yerkes-Dodson curve [27], performance levels may decline when levels of stress exceed the optimal point on the curve. Beyond this optimal level of stress, stressors are likely to be perceived as overwhelming or excessive and individuals may appraise these stimuli in a more negative light, resulting in them questioning whether they have the necessary resources to cope effectively. In this review, when using the term ‘stress,’ or when focusing on the “stress response”, we are referring to times whereby individuals are experiencing negative effects or interpretations of stress (i.e., distress).

Poorly managed, overwhelming stress can result in exhaustion (i.e., last stage of the GAS model), psychological breakdown (e.g., depression, anxiety, insomnia) and other detrimental health consequences (e.g., high blood pressure, weakened immune system and diseases that adversely affect the cardiovascular, neuroendocrine and central nervous systems) [32,33,34,35,36]. The significance of these implications is underscored by the prevalence of stress among the population; as many as 35 percent of Australians have reported significant levels of stress in their lives at a single time point [37]. More alarmingly, about 75% of adults reported moderate to high levels of stress—most likely in the form of distress—in the 2014 Stress in America survey [38]. Besides affecting many adults, stress is also a pervasive health concern for teenagers and stress among adolescents may contribute to serious long-term health implications if not adequately addressed [39].

## 4. Effect of Stress on Eating Behaviour and Food Choices

Stress can directly increase the risk of chronic diseases and health problems but can also indirectly influence these outcomes via its effects on health-related behaviour [40,41]. For instance, sleep habits, substance use (including alcohol and smoking) and food choices are behaviours often negatively affected by stress [42]. For instance, stress is considered one of the major contributors to the initiation and continuation of alcohol and drug use as well as addiction relapse [43,44]. Cole and colleagues [45] found that life stressors and perceived levels of stress were positively correlated with alcohol drinking behaviour. Despite the significant and detrimental impact of stress on alcohol consumption and drug use, the primary focus of this present paper is on the influence of stress on food intake and food choices. With reference to the relationship between stress and food intake, 30% of respondents in the 2013 Stress in America survey reported skipping a meal when stressed, with 41% of the people who skipped meals reporting that they did so at least on a weekly basis [39]. Interestingly, the results of the same survey showed that 38% of adults reported overeating or increasing the consumption of unhealthy food because of stress; and approximately half of these adults (49%) reported engaging in these behaviours once or more each week [39]. In further support of a relationship between stress and (potentially unhealthy) food intake, it was reported in the 2015 Stress and Wellbeing Survey that 75 percent of Australians turned to eating as a stress coping mechanism [37].

As reflected in the above statistics, there appears to be individual variation in terms of the exact nature of the relationship between stress and food intake. Some investigators have reported that individuals report or display higher caloric intake during stressful periods [46,47,48,49], others have failed to find any overall difference in energy intake between stressful and non-stressful situations [50,51,52] and some have reported decreases in appetite and food intake when exposed to an acute stressor [53]. The reasons for this are likely multi-factorial and with factors such as gender, body mass index, restraint in eating [54] and even the time of the day [55] potentially moderating stress-induced eating. Despite the discrepancies reported in previous studies regarding the overall amount of energy intake in response to stress, researchers typically report a shift in food choices—in both humans and animals—toward foods high in fat and sugar content under stressful conditions. In animal studies, it has been observed that animals increase food intake when in a mildly or moderately stressful environment and when provided with access to highly palatable food that is high in fat and sugar [56,57,58]. This observation is consistent with human studies examining food choice in response to stress [59,60,61,62]. Indeed, when provided with choice, foods consumed during times of both chronic and acute stress typically favour those that are energy-dense, with increased fat and/or sugar content [63,64,65]. This shift in food choice, toward the consumption of more pleasurable or palatable foods in stressful situations, appears to occur regardless of alterations in the total caloric intake [6,66,67,68,69,70]. This phenomenon is also observed under controlled laboratory circumstances, with an increase in the intake of “comfort” foods when both humans and animals are placed under acute physical or emotional stressors [64,65,70,71,72,73,74], even when individuals have high satiety and have no homeostatic need for calories [64,75]. Accordingly, there is growing evidence that although stress may have varied effects on overall food intake, the types of foods consumed may be skewed toward the preferential consumption of unhealthy energy-dense foods and drinks [5,48].

### 4.1. Stress-Induced Eating: Potential Physiological Mechanisms

The precise mechanisms through which stress influences food intake are likely multi-faceted and may be both physiological and psychological in nature. With respect to physiological mechanisms, two key hormones—namely cortisol and ghrelin—may play a significant role. Stress activates the hypothalamic-pituitary-adrenal (HPA) axis, ultimately resulting in the release of corticosteroids, which are known to stimulate appetite [76]. In an acute setting, cortisol has been shown to peak 10 to 20 min after a stressor is removed [77,78,79], before returning to near baseline in approximately one hour [80,81]. Meanwhile, peak cortisol release has been reported to correspond with ad libitum intake of snack foods [82], while daily cortisol administration has been shown to increase total daily ad libitum energy intake in healthy men [83]. With specific respect to a stress-induced increase in cortisol, Epel and colleagues [71] found that women who secreted more cortisol in response to a laboratory stressor consumed more food (particularly sweet foods) while recovering from stress, compared with participants who were low cortisol responders. However, it is important to note that other researchers have not found an association between stress-induced cortisol and increased food intake [52,84]. The reason for these mixed findings is unclear but may be due to individual differences in the response to stress, or the presence of chronic stress. Continued exposure to stressors can result in an accumulation of plasma cortisol [85], which may lead to increased appetite [83]. Intake of highly palatable food may in turn decrease the activity of the HPA axis, therefore dampening the stress response itself, which may in turn serve to reinforce comfort eating when individuals are stressed. This notion of a feedback loop is supported by reductions in cortisol [62] and corticosterone levels [59,61,62] in response to intake of highly palatable foods after exposure to stressors. Yet, there is also evidence that chronically stressed individuals may respond to an acute stressor with a dampened cortisol rise (i.e., hypo-activity of the HPA axis) [86]. Furthermore, this blunted cortisol response to acute stress has been associated with unhealthy food intake and choices [87,88], especially in highly emotional eaters [89]. Clearly, more research is needed to better understand the interaction between chronic and acute stress, cortisol and appetite.

Besides cortisol, ghrelin may play a role in stress-induced eating. Ghrelin, also known as the “hunger hormone” [90], is found to rise immediately before eating and fall again within the next hour [91]. With respect to stress, Rouach and colleagues [92] found that an acute psychological stressor (i.e., the Trier Social Stress Test; [93]) induced an increase in plasma ghrelin levels. This stress-induced increase in plasma ghrelin positively correlated with the acute response of serum cortisol to the stressor and individuals in the higher quartile of ghrelin response also reported higher subjective scores for psychological stress. However, self-reported compulsion to eat did not differ according to ghrelin response [92]. Nonetheless, this stress-induced rise in ghrelin has also been reported by others [94,95]; and whether these hormonal fluctuations actually translate into changes in eating behaviour and unhealthy food choices requires further investigation.

It should also be acknowledged that the hedonic reward system may play a role in stress-induced eating. The theoretical model of Reward Based Stress Eating [59] proposes that stress activates the HPA axis, which in turn activates the brain reward system, leading to the release of endogenous opioids which may increase the motivation to seek palatable food. Meanwhile, there is some evidence that acute stress decreases the sensitivity of reward areas of the brain to food cues [4], suggesting that increased intake of palatable food may be sought in order to obtain the usual reward. However, more research is needed in this area to enhance our understanding of the potential role of the brain reward system in stress-induced eating.

### 4.2. Stress-Induced Eating: Potential Psychological Mechanisms

In addition to prominent physiological control of appetite, it is known that psychological factors underpin eating behaviour [66,96]. Stress often leads to negative emotions such as anxiety and depression [97,98] and it is well known that such negative emotions can give rise to unhealthy eating [5,99,100]. Furthermore, researchers have found that foods chosen to cope with these negative emotions are often energy-dense and particularly high in sugar and fat [101,102]. While some argue that emotional eating might not effectively regulate negative emotions but instead increases them [103], others propose that emotional eating is often driven by hedonism, which reflects the desire to increase and maintain the experience of positive emotional states [59,104]. This in turn may contribute to recurring nature of emotional eating, with individuals who turn to unhealthy and palatable food choices each time they are exposed to stressful situations experiencing behavioural reinforcement [63], resulting in the establishment of “comfort eating” in response to stress [105].

Another psychological factor that may influence an individual’s ability to make healthy food choices when exposed to stress relates to “ego depletion”. According to proponents of the strength model of self-control (e.g., [106]), one’s capacity to override natural impulses and automatic or habitual responses is viewed as a limited intra-individual resource that becomes depleted with use. As a result, acts of self-control may diminish one’s capacity to enact further acts of self-control and a state of ego depletion is experienced when self-control is exhausted. Individuals in this state (or those who have had their self-control depleted to a greater extent than a comparison group) have been shown to spend more money impulsively [107], perform inappropriate sexual behaviours [108], respond with higher levels of aggression [109] and drink more alcohol even when anticipating a driving test [110]. Ego depletion has also consistently been found to influence food choices, such that individuals in this state consume more tempting, palatable food compared with control participants (e.g., [111,112]). Researchers have also found that people are more prone to breaking diets when ego depleted [107] and several studies have shown that restrained eaters consume more following engagement in a self-regulation task than non-restrained eaters [107,113]. Therefore, given that coping with stress may involve processes that demand inhibition, such as ignoring sensations, overriding negative thoughts, suppressing emotions and attention regulation [114,115], stressful events might draw from and deplete individuals’ limited pool of self-control resources and accordingly, make individuals susceptible to the impulsive consumption of palatable, unhealthy food. 

## 5. Stress Management

Given the adverse health implications associated with stress, including stress-induced eating, it is important that effective ways to manage stress are identified. Strategies to cope with stress may be broadly considered problem- or emotion-focused in nature [23]. Problem-focused coping strategies are characterized by individuals changing, or modifying, the fundamental source of stress, with the overarching goal to reduce or remove the cause of the stressor. Examples of problem-focused coping strategies include taking control (e.g., time management, goal setting) and information seeking (e.g., utilizing problem-solving skills, advice seeking, learning new skills). Problem-focused coping may not always be practical or optimal, however, such as when a stressor is unchangeable or unmodifiable (e.g., the death of a loved one). In such instances, individuals may employ emotion-focused coping strategies [116,117], which involve self-reflection and taking control over one’s emotions [118], with the aim of changing the meaning of the stressor (i.e., reappraisal) or transferring attention away from it (i.e., avoidance) [119]. Many evidence-based techniques, such as relaxation therapy (e.g., breathing exercises, muscle relaxation) and meditation (e.g., contemplation, mindfulness), which bring about inner awareness and calm, represent emotion-focused coping techniques. Other beneficial emotion-focused coping strategies include listening to music, seeking social interaction and support, reading, exercising and reinterpreting one’s situation in an attempt to view it in a more positive light [37].

There are also forms of emotion-focused coping that may be considered negative or harmful in nature, such as using gambling, smoking, alcohol, and/or recreational drugs as a method for avoiding or distracting oneself from a stressor [37]. In particular, stress is highly predictive of both alcohol use and drinking problems among men who rely on avoidant forms of emotion-focused coping [44]. Relevant to the present discussion, stress-induced eating (particularly the consumption of unhealthy foods) may also represent a maladaptive coping strategy used by some; indeed, 45% of respondents in a recent Australian survey reported using eating as a way to manage stress even though they indicated that eating was not an effective coping mechanism [37]. It is clearly important for long-term health and wellbeing that individuals implement adaptive emotion-focused coping strategies rather than those that may stimulate additional health-related or other stressors (e.g., financial hardship). Among those factors that are considered adaptive in nature, it is well established that one of the most effective way of managing stress is through participation in physical exercise. In the Stress in America survey 2013, 43% of adults identified that they used exercise as a method to manage stress and many reported that they experienced associated benefits in terms of elevated mood, more positive self-concept and stress reduction when they exercised [39]. In the following section, we present evidence for how physical exercise may protect against psychological and physiological stress responses and consider, in particular, how exercise may operate with respect to the stress models previously outlined.

## 6. Effect of Physical Exercise on Stress

Broadly speaking, there is substantial research evidence to show that physical exercise is protective against detrimental stress responses [120,121,122,123]. With respect to psychological well-being, intervention and prospective studies have demonstrated that regular exercise reduces perceived stress in real-world settings [122,123,124]. Moreover, randomized clinical trials have shown that both regular exercise participation and acute bouts of exercise are effective in reducing perceived stress and improving quality of life [13,14,15,125,126,127,128,129]. For instance, Aldana and colleagues [130] reported that, compared with individuals who did not exercise, those who expended more than 3.0 kcal/kg/day in physical activity were 78% less likely to experience moderate stress and 62% less likely to experience high perceived stress. These researchers also found that individuals who participated in exercise of moderate intensity exhibited approximately half the amount of perceived stress as those who reported no exercise [130]. In addition to the benefits of exercise for reducing perceived stress, regular exercise may also ameliorate the physiological responses to both acute and chronic stress. Individuals who adhere to regular exercise have been shown to exhibit smaller increases in diastolic blood pressure following exposure to psychological stressor when compared with those who are less physically active [131,132]. There is also evidence that an acute bout of exercise of moderate to vigorous intensity (60–75% maximal oxygen uptake or 75% heart rate reserve) performed prior to exposure to a stressor appears to attenuate the blood pressure response to stress [15,133,134,135]. Further, Brownley and colleagues [136] found that the attenuation of the post-exercise blood pressure in response to stress was associated with a reduction in the response of catecholamine to stress.

## 7. Effect of Physical Exercise on Stress-Induced Eating

Even though physical exercise appears to be an effective strategy for coping with stress, limited research attention has been directed toward examining whether exercise can attenuate stress-induced eating. Furthermore, the specific mechanisms through which exercise may reduce unhealthy eating in response to stress are yet to be determined. Here we present the empirical work examining the effect of exercise and stress on appetite-related variables to date (summarized in Table 1), before we consider how exercise might influence the physiological and psychological pathways through which stress appears to increase the likelihood of consuming unhealthy energy dense foods and drinks.

### 7.1. Evidence for an Effect of Physical Exercise on Stress-Induced Eating

Taylor and Oliver [137] conducted the first study to address this issue. They found that a 15-min bout of brisk-walking performed just prior to a stressor (albeit potentially a relatively minor one—a Stroop colour-word interference task) did not significantly alter cravings for chocolate when cued compared to a no-exercise control in normal-weight men and women who were regular chocolate consumers. However, these researchers did report moderate effect sizes, suggesting a trend toward an attenuated response to the chocolate cue following exercise compared with a no-exercise condition. In a more recent study, Ledochowski and colleagues [138] found that a 15-min bout of brisk-walking performed before exposure to a stressor (again a Stroop colour-word interference task) reduced subsequent cravings for sugary snacks compared with a control condition without physical exercise in overweight men and women. The difference between the findings reported by Ledochowski and colleagues [138] and those of Taylor and Oliver [137] may be related to the nature of the participants studied (i.e., overweight compared with normal-weight individuals). It is possible that short bouts of physical activity have a stronger effect in reducing the urge to consume food among overweight participants compared with normal weight participants, especially at times when the person may be particularly vulnerable such as during stressful episodes and when snack foods are available.

In a separate study, Oh and Taylor [139] examined the effects of a 15-min bout of brisk-walking on ad libitum chocolate consumption during exposure to a stressor (Stroop colour-word interference task) in normal weight chocolate eaters. Interestingly, they found that rested participants ate almost twice as much chocolate as those who exercised, suggesting that a brief bout of physical activity may reduce ad libitum eating while performing stressful mental tasks. However, it is important to highlight that, in this study, chocolate was provided during exposure to the stressor and not afterwards. It is also worth noting that a 15-min exercise bout and the Stroop colour-word task were employed in each of the examples described above. As evidence develops in this field, it would be beneficial to test whether longer duration and higher intensity exercise may have more substantial (or different) effects on stress-induced eating and whether activities other than the Stroop task are more effective at inducing genuine “stress” responses (as outlined in the transactional model; [140]).

In one example of such an approach, Horsch and colleagues [141] examined the effect of 30 min of moderate intensity exercise prior to acute stress exposure on food intake and choice in overweight and normal weight children. Children were randomly allocated to perform physical exercise (moderate-intensity) or sedentary activity for 30 min before exposure to the Trier Social Stress Test for children [142]. Participants were then allowed to eat ad libitum from a range of low to high caloric, salty or sweet foods. These researchers reported that prior exercise was associated with a significant reduction in the intake of low-caloric salty foods and a tendency for lower overall carbohydrate intake. In another recent example in which “stress” was induced using a method other than the Stroop task, Neumeier and colleagues [143] reported that a bout of 15-min bout of high intensity (80–85% maximal oxygen uptake) interval exercise could prevent a surplus of energy intake when performed after stressful mental work. The mental work consisted of a sample of graduate entrance examination level reading comprehension problems and one college entrance level math problem. Participants were instructed to try their best and were given 20 min to complete as much of the problem set as possible. Participants were randomly allocated into 2 different groups (“mental work with rest” or “mental work with exercise”) and completed their respective conditions as well as an additional baseline rest condition, each of which were followed by access to a standardized high-caloric meal (pre-made pizzas) from which they could eat ad libitum for 30 min. The researchers reported that participants who did not participate in physical exercise consumed significantly more calories after mental work compared to their rest condition. In sum, although these studies provide some promising preliminary evidence for a potential role of exercise to attenuate stress induced eating, the physiological and psychological pathways through which exercise achieves this effect remains to be determined.

### 7.2. Potential Physiological Mechanisms through Which Exercise May Attenuate Stress-Induced Eating

**Figure 1:**
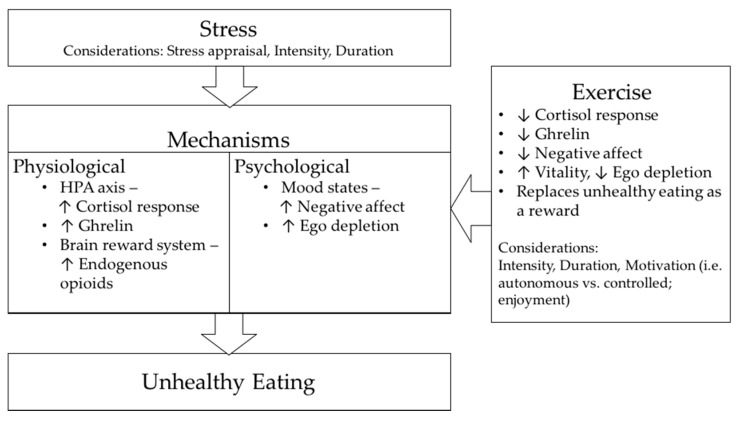
Potential mechanisms by which exercise might attenuate stress-induced eating.

One possible mechanism by which exercise may attenuate unhealthy food intake in response to stress relates to the effect of exercise on the HPA axis, especially the circulating cortisol response to stress. As previously outlined, the release of cortisol is found to stimulate appetite in the recovery period following stress [76]. Hence, by influencing cortisol levels, it is possible that physical exercise may act on the resistance stage of the GAS stress model and therefore play a role in reducing unhealthy food consumption in response to stressor exposure. In support, a number of studies have demonstrated lower plasma cortisol levels, or reduced pituitary sensitivity to glucocorticoids in endurance-trained individuals compared with their sedentary counterparts [144,145,146,147]. With specific respect to stress, Traustadottir and colleagues [148] reported that the cortisol response to a standardized psychosocial stressor was substantially lower in women with higher aerobic fitness compared with women with lower aerobic fitness. Meanwhile, Zschuke and colleagues [16] found that an acute bout of moderate intensity exercise performed before a psychological stressor significantly reduced the subsequent cortisol response. Together, these findings indicate that both acute and regular exercise may result in a higher stressor intensity being required to induce a given cortisol response. How this might influence subsequent food intake remains to be determined. Besides the potential role of exercise in reducing the cortisol response to stress, there is also evidence that an acute bout of exercise performed at moderate intensity results in a transient suppression of the circulating levels of acylated ghrelin [149,150,151]. Whether this effect is maintained under conditions of stress and how it may affect the downstream effects of unhealthy food consumption requires investigation. Figure 1 below summarises the potential mechanisms by which exercise might attenuate stress-induced eating.

### 7.3. Potential Psychological Mechanisms through Which Exercise May Attenuate Stress-Induced Eating

In addition to acting on these potential physiological mechanisms, exercise may also serve to modify the psychological processes responsible for stress-induced eating. As outlined, when a stressor is appraised as a threat or a harm-loss, individuals may experience negative affect and the desire to attenuate negative affect may then drive hedonically pleasurable (e.g., unhealthy) food consumption. Research evidence indicates that negative mood states, such as depression and anxiety, are reduced following a single episode of exercise and these effects may last from several hours to a full day [152,153,154]. In one relevant example, Steptoe and colleagues [155] found that a 10-week exercise intervention of moderate intensity resulted in significant reductions in tension-anxiety and depression after a series of mental stress tests, together with an increased perceived ability to cope with stress (i.e., influencing the secondary appraisal process within the Transactional Model) as compared with a placebo training intervention. Moreover, it has been reported that both acute and regular bouts of aerobic exercise can reduce stress-induced negative affect [156,157] and may, therefore, contribute to attenuating the affective processes that may be partly responsible for stress-induced eating. Exercise also activates brain reward systems [158,159], which play an important role in behaviour reinforcement. Consequently, exercise has been suggested as a potential treatment approach (i.e., as a form of replacement) for individuals with drug or substance addiction [160,161,162]. Taken together, it appears plausible that individuals who may otherwise turn to unhealthy, palatable food consumption (or even to other unhealthy substances, such as alcohol or drugs) as a “reward” when they are stressed, may feel less compulsion to do so if they have previously exercised. 

As discussed earlier, one’s ability to make healthy food choices may also be determined, at least in part, by an individual’s level of ego depletion. Given that acts of self-control are ego depleting, it is possible that one’s motivation toward exercise may play a role in influencing responses to post-exercise stressors that are dependent upon ego depletion status [163,164,165,166]. More specifically, it is possible that individuals’ reasons (or motives) for exercise may render them more or less susceptible to failures of self-control (e.g., unhealthy, pleasurable food intake) when faced with a stressor following exercise. Within self-determination theory (SDT), Deci and Ryan [167,168] articulate that motivation can be understood not only in terms of the quantity (or level) of motivation that someone possesses but also in terms of the quality of that motivation. Broadly, within SDT it is theorized that individuals may experience autonomous, or self-determined, motives for an activity, as well as more controlled (less self-determined) motives. In the case of exercise participation, an autonomously motivated individual may pursue the activity because he or she finds it inherently fun and interesting, values the outcomes associated with it, and/or feels that exercise aligns with his or her identity and sense of self. In contrast, a person who experiences relatively greater controlled motivation for exercise might participate in the activity because he or she feels guilty or ashamed for being inactive, is pressured to do so by another person, and/or is simply trying to obtain some external reward (e.g., monetary, praise, etc.).

When people’s motivation for exercise is governed primarily by controlled (as opposed to autonomous) reasons, exercise is more likely to be ego depleting. To illustrate, we might consider a person who runs during his/her lunch hour at work. On the one hand, this person might simply enjoy running and value it (and exercise more broadly) for the way it makes him/her feel. On the other hand, this person may not consider him/herself an “exerciser” and might be exercising simply because his/her partner, family, or medical practitioner has told him/her that s/he has to exercise to become healthier. In the former example, due to the motivational considerations underpinning the activity, there is likely to be little self-control, or willpower, required in regulating the exercise and executing the movements (i.e., there is no impulse to override). Moreover, the exercise itself might be viewed as vitalizing and contribute to the restoration of mental “reserves” (i.e., self-control resources). In the latter example, however, it is more likely that the person has to employ at least some degree of willpower in order to go for the run and as a result, might finish his/her exercise with lower self-control (than the autonomously motivated exerciser) and be more susceptible to subsequent failures in self-control. Theoretically speaking, exercise participation might act on stress-induced eating through a self-control mechanism; and, more specifically, the motivational regulations that underlie any exercise prior to facing a stressor may shape the amount of self-control available to the self (whereby controlled exercise motivation might induce ego depletion more so than autonomous exercise participation). In turn, these self-control levels might subsequently be responsible for determining one’s ability to override impulses associated with unhealthy, pleasurable food following a stressor exposure.

In support of this notion, there is evidence indicating that an individual’s autonomous regulation of behaviour (unrelated to food consumption) is indeed positively associated with healthier food choices [169,170,171]. Specifically with respect to the relation between exercise motivation and post-exercise food consumption, West and colleagues [172] demonstrated that individuals who reported experiencing greater controlled (relative to autonomous) motivation were more likely to endorse (i.e., license themselves regarding) the consumption of unhealthy snacks and drinks following their participation in an acute exercise session. In another recent study, in which actual energy intake was assessed, Beer and colleagues [173] found that a lack of choice in exercise—which, theoretically speaking, should encourage more controlled, rather than autonomous experiences—was associated with greater energy intake from unhealthy foods. According to SDT, autonomous motivation is promoted when social-contextual conditions support, among other things, individuals’ basic psychological need for autonomy (i.e., choice, volition, self-regulation) and so it is possible that choice in exercise—and the resultant beneficial downstream motivational outcomes—may help to attenuate the effects of post-exercise stressors on food intake and choices. Accordingly, it would be worthwhile in future investigations to replicate the work described above, with the additional design element of exposure to a stressor post-exercise, in order to more comprehensively test the pathways being presently proposed.

## 8. Conclusions

In this article, we have outlined stress as a concept and considered how, in the various stress models, both acute and chronic stress can be detrimental to our health and wellbeing. Stress not only affects our health directly via increasing the risk of chronic diseases and health problems but also indirectly by influencing these outcomes via its effects on health-related behaviour—in particular, stress-induced eating. With reference to the potential physiological and psychological pathways that contribute to unhealthy eating following stress exposure, there exists preliminary supporting evidence for a role of exercise in attenuating these responses. Despite substantial research attention being directed to stress and exercise, limited studies have examined whether the effects of physical exercise on the acute stress response can further influence stress-induced eating and deter unhealthy food choices; and the mechanisms by which this may be achieved. Further, with the effects of physical exercise on the stress response and implications for subsequent food consumption being multi-faceted, more research is needed to test potential moderating factors—in particular exercise motivation. Indeed, further research is warranted to test for this and other potential moderating factors, that might influence the interaction between exercise, stressor exposure and food choices.

## Figures and Tables

**Table 1:** Previous literature investigating the effects of physical exercise and stress on appetite-related variables.

Authors (Year)	Participants	Experimental Design	Intervention	Stressor	Appetite-Related Variable	Results
Taylor & Oliver (2009) [137]	25 normal weight, regular chocolate eaters	Within subjects	Ex (15 min brisk walking) vs. Con (15 min quiet sitting) (pre-stressor)	Stroop colour-word interference task	Chocolate cravings	Exercise did not significantly reduce cravings (*p* = 0.06, moderate effect sizes)
Ledochowski et al. (2015) [138]	47 overweight sugary snack eaters	Within subjects	Ex (15 min brisk walking) vs. Con (15 min quiet sitting) (pre-stressor)	Stroop colour-word interference task	Sugary snack cravings	Exercise significantly reduced cravings (*p* < 0.01)
Oh & Taylor (2012) [139]	78 normal weight, regular chocolate eaters	2 × 2 Factorial design	Ex (15 min brisk walking) vs. Con (15 min quiet sitting) (pre-stressor)	Stroop colour-word interference task (low and high demanding)	*Ad libitum* chocolate consumption	Exercise significantly reduced consumption after both low and high demand stress conditions (*p* < 0.01)
Horsch et al. (2015) [141]	26 normal weight (NW), 24 overweight (OW) children	2 × 2 Factorial design	NW Ex (30 min moderate intensity exercise) vs. NW Con (sedentary) vs. OW Ex (30 min moderate exercise) vs. OW Con (pre-stressor)	Trier Social Stress Test for children	*Ad libitum* food consumption	Exercise significantly reduced low-caloric salty food intake (*p* < 0.001) and tendency for lower overall carbohydrate intake (*p* = 0.07)
Neumeier et al. (2016) [143]	38 normal weight university students	Between groups (with each group compared to their baseline rest)	Ex (15 min high intensity interval exercise) vs. Con (rest) (post-stressor)	Graduate entrance level reading comprehension problems and one college entrance math problem	*Ad libitum* pizza consumption	Con consumed significantly more calories compared to baseline rest (*p* = 0.02) but EX did not increase intake (*p* > 0.05)

Note: Ex—Exercise condition, Con—Control condition.
